# Progression of Fetal Brain Lesions in Tuberous Sclerosis Complex

**DOI:** 10.3389/fnins.2020.00899

**Published:** 2020-08-21

**Authors:** Antoinette Bernabe Gelot, Alfonso Represa

**Affiliations:** ^1^Aix-Marseille University, INSERM, INMED, Marseille, France; ^2^APHP, Hôpital Trousseau, Université Pierre et Marie Curie, Paris, France

**Keywords:** tubers, dysmorphic neurons, Giant cells, development, interneurons

## Abstract

Tuberous sclerosis (TSC) is a multisystem autosomal dominant genetic disorder due to loss of function of *TSC1/TSC2* resulting in increased mTOR (mammalian target of rapamycin) signaling. In the brain, TSC is characterized by the formation of specific lesions that include subependymal and white matter nodules and cortical tubers. Cells that constitute TSC lesions are mainly Giant cells and dysmorphic neurons and astrocytes, but normal cells also populate the tubers. Although considered as a developmental disorder, the histopathological features of brain lesions have been described in only a limited number of fetal cases, providing little information on how these lesions develop. In this report we characterized the development of TSC lesions in 14 fetal brains ranging from 19 gestational weeks (GW) to term and 2 postnatal cases. The study focused on the telencephalon at the level of the caudothalamic notch. Our data indicate that subcortical lesions, forming within and at the vicinity of germinative zones, are the first alterations (already detected in 19GW brains), characterized by the presence of numerous dysmorphic astrocytes and Giant, balloon-like, cells. Our data show that cortical tuber formation is a long process that initiates with the presence of dysmorphic astrocytes (by 19–21GW), progress with the apparition of Giant cells (by 24GW) and mature with the appearance of dysmorphic neurons by the end of gestation (by 36GW). Furthermore, the typical tuberal aspect of cortical lesions is only reached when bundles of neurofilament positive extensions delineate the bottom of the cortical lesion (by 36GW). In addition, our study reveals the presence of Giant cells and dysmorphic neurons immunopositive for interneuron markers such as calbindin and parvalbumin, suggesting that TSC lesions would be mosaic lesions generated from different classes of progenitors.

## Introduction

Tuberous sclerosis (TSC) is a systemic, autosomal dominant genetic disorder caused by mutations of *TSC1* or *TSC2* genes (Dragoumi et al., 2018; Hasbani and Crino, 2018) that result in a constitutive activation of mTORC1 disturbing subsequently cellular differentiation, proliferation, and migration early in development. Brain TSC lesions, found in around 90% of TSC patients, have been well characterized and include cortical tubers and white matter and subependymal nodules (Mizuguchi and Takashima, 2001; Aronica and Crino, 2014), where dysmorphic neurons and Giant cells can be considered as histopathological hallmarks.

Giant cells are large, distorted, ballooned cells with a rather flattened and decentered nuclei and opalescent cytoplasm similar to Balloon cells described by Taylor et al. (1971) in focal cortical dysplasia. They are mainly present in deep layers of the affected cortical area and corresponding white matter (Taylor et al., 1971). Giant cells express markers of neural progenitors (SOX2, NESTIN, VIMENTIN and CD133) (Garbelli et al., 1999; Urbach et al., 2002; Ying et al., 2005; Lamparello et al., 2007; Yasin et al., 2010; Prabowo et al., 2013) and express in some cases markers of mature astrocytes (GFAP, S100β) or neurons (neurofilament, NeuN, Tuj1, synaptophysin) (Garbelli et al., 1999; Urbach et al., 2002; Ying et al., 2005; Prabowo et al., 2013). From these observations it has been proposed that Balloon cells/Giant cells might derive from radial glial/stem cells (Lamparello et al., 2007; Yasin et al., 2010). However, it cannot be excluded that some of them derive from more restricted progenitors’. Giant cells in tubers are associated with a clear disorganization of cortical layering and the presence of dysmorphic neurons and reactive astrocytes. The later may result from immune-inflammatory responses (for review see Aronica and Crino, 2014), though it was suggested that astrogliosis would result from the initial mutation of TSC1/TSC2 genes as many reactive astrocytes in the tubers express increased immunoreactivity for activated mTOR pathway components such are phospho-p70S6 kinase, phospho-S6 and phospho-STAT3 (Sosunov et al., 2008).

TSC lesions have been identified in fetal brain samples but so far only a few cases have been evaluated for cellular components and lesion changes: two cases at week gestational age (GW) 24 and 30 by Bordarier et al. (1994); one case at 20GW case by Park et al. (1997) and six cases of 23, 27, 32, 34, and 38 GW by Prabowo et al. (2013). From these studies it can be proposed that (i) TSC lesions are present as early as 20GW. (ii) In the earliest stages (20–27GW) TSC lesions are mainly subcortical while they appear in the cortical plate from 32GW. (iii) Fetal Giant cells do not express neuronal markers. (iv) Dysmorphic neurons are not observed in fetal cases. Together these published data support the notion that the presentation and features of tuberal lesions is dependent on the developmental stage and that lesions evolve with the cortical development. In this report, we propose to revisit these notions by analyzing 16 cases from GW 19 to the 8th postnatal month. Our data confirm the early presence of subcortical TSC lesions (mainly subependymal and white matter nodules, already present in 19GW samples). Interestingly, they reveal the presence in subcortical lesions of dysmorphic neurons and Giant cells expressing neuronal markers (by 34GW and 26GW respectively). Third, we also confirm the early onset of cortical lesion (by 21GW) but our data demonstrate that the acquisition of the typical cortical TSC features is a rather late event, when dysmorphic neurons and abnormal bundles of neurofilaments appear (by 36GW). Finally, our data uncover for the first time a potential involvement of inhibitory interneurons lineage in the formation of TSC lesions, reinforcing the notion that TSC lesions are mosaic lesions arising from different progenitors classes.

## Materials and Methods

This retrospective study includes specimens obtained from the brain collection “Hôpitaux Universitaires de l’Est Parisien – Neuropathologie du développement” (Biobank identification number BB-0033-00082) (Table 1). For all the cases studied, informed consent was obtained for autopsy of the brain and histological examination. The study included 16 patients for whom the pathological diagnosis of TS was made prenatally or confirmed after the post-mortem examination. The study also includes three control fetal brains (36–37GW) obtained from spontaneous abortion that did not display any significant brain pathology.

**TABLE 1:** Clinical, neuroimaging, neuropathological and molecular findings of TSC cases.

**—Case**	**GW or postnatal day or month**	**Gene**	**Prenatal features (US and MRI findings)**	**Macroscopic pathological lesions**	**Histological lesions**		
					**SEN**	**WMN**	**Cortical tuber**
1	19GW	ND	Familial polycystic kidney disease (Cardiac Rhabdomyome at autopsy)	No apparent lesions	+ (parietal)	+ (deep only)	−
2	21GW	TSC2	Intraventricular hemorrhage	Contralateral Hemimegalencephaly + SEGA	+ (ganglionic eminence)	+ (deep only)	−
3	25GW	TSC2	Cardiac Rhabdomyome	SEN (parietal at the caudothalamic groove), Abnormal gyration (frontoparietal)	+ (caudothalamic groove)	+	−
4	25GW	ND	Multiple cardiac Rhabdomyome	No apparent lesion	+ (caudothalamic groove)	+	−
5	26GW	TSC2	Cardiac Rhabdomyome	SEN (temporal and parietal at the caudothalamic groove), Abnormal gyration (parietal)	+ (caudothalamic groove)	+	−
6	30GW	ND	Cardiac Rhabdomyome	Abnormal gyration	+ (caudothalamic groove)	+	−
7	30GW	ND	Cardiac Rhabdomyome	No apparent lesion	+ (caudothalamic groove)	+	−
8	32,5GW	ND	Cardiac Rhabdomyome	Abnormal gyration	+ (caudothalamic groove)	+	−
9	34GW	TSC2	Cardiac Rhabdomyome + CNS lesions	SEN (temporal and parietal at the caudothalamic groove), WMN	+ (caudothalamic groove)	+	−
10	34GW	TSC2	Cardiac Rhabdomyome + CNS lesions	SEN (temporal and parietal at the caudothalamic groove), WMN	+ (caudothalamic groove)	+	−
11	35GW	TSC2	Cardiac Rhabdomyome + CNS lesions	Exuberant SEN (temporal and parietal at the caudothalamic groove), WMN	+ (caudothalamic groove)	+	−
12	36GW	Unaltered TSC1 and TSC2	Cardiac Rhabdomyome + CNS lesions	Exuberant SEN (temporal and parietal at the caudothalamic groove), WMN, Cortical Tuber	+ (caudothalamic groove)	+	+
13	36GW	TSC2	Cardiac Rhabdomyome + CNS lesions	SEN (temporal and parietal at the caudothalamic groove), WMN, Cortical Tuber	+ (caudothalamic groove)	+	+
14	39GW	ND	Cardiac Rhabdomyome + CNS lesions	SEN (temporal and parietal at the caudothalamic groove), WMN, Cortical Tuber	+ (caudothalamic groove)	+	+
15	3PND	ND	Cardiac tumor and hearth failure	Subarachnoid hemorrhage, SEN and WMN	+ (caudothalamic groove)	+	+
16	8 PM	ND	Status epilepticus	SEN (parietal at the caudothalamic groove) WMN, Disseminated Cortical tuber	+ (caudothalamic groove)	+	+

*CNS lesions: focal MRI hypersignal in fetal brain/gyration disturbance/Periventricular tuber.*

We applied the same technical procedures to all brains studied, patients or control. After removal, brains were fixed with formalin for 5–12 weeks. Macroscopic analysis was performed allowing the selection and conditioning of samples (paraffin embedding, 7-micron slicing, hematein staining) of brain tissue for histological analysis.

Immunohistochemical analyses on cases 1, 3–4, 6–8, 10–15 were performed with an automat (LEICA BOND III) on one or two coronal slices at the caudothalamic notch that included the fronto-parietal telencephalic parenchyma. The following antibodies (see Supplementary Table 1) were used for Giant cell and glial staining: mouse anti-vimentin (clone V9, RTU Leica Microsystem), Mouse anti-GFAP (clone GA5, RTU Leica Microsystem), Rabbit Anti-Glutamine Synthetase antibody (1:5000, Abcam ab49873); for neuronal staining: goat polyclonal Anti-Doublecortin antibody DCX (1:600 Abcam, ab113435), mouse anti NF200 (Neurofilament 200 kD clone N52.1.7 Leica Biosystem PA0371), *mouse* anti-NeuN (1:50, clone A60; Millipore MAB377) and for interneurons: Mouse anti-calretinin (clone CAL6, RTU Leica Microsystem), rabbit anti-GABA (1:500; Sigma A2052), mouse anti reelin (clone G10, 1:400 Chemicon MAB5364) mouse anti calbindin (clone KR6 1:100 Leica Microsystem) and sheep anti-parvalbumin (1:320 R&D systems). We used anti-Ki67 (clone MM1, RTU Leica Microsystem) and rabbit anti PS6 (1:100, clone 4857, Cell Signaling Technology, Danvers). Finally, we also used rabbit anti FLNA (1:250 clone EP2405Y; Abcam ab76289), as a recent study reported that filamin A antibodies stained Giant/Balloon cells and dysmorphic neurons in samples from TSC and FCD patients (Zhang et al., 2020). Primary antibody binding to tissue sections was visualized using BOND Polymer Refine Detection (Leica Microsystem) except for parvalbumin (1:100 secondary horseradish peroxidase-conjugated IgG anti-sheep, Abcam). Images were acquired using an Olympus BX40 microscope (Olympus, Tokyo, Japan) equipped with a color Q imaging camera (1600 × 1200 pixels).

Measures of parvalbumin + and calbindin + cortical cells were performed using ImageJ 1.52q, on 40x acquisitions. The analysis included the cortical tuber and an equivalent area with no apparent TSC. For comparison, we analyzed samples from three age-matched (36–37GW) control cases without neuropathological alterations. Statistical analyses were performed with using Prism 6 (Graphpad). Normality of the data distributions was systematically tested using d’Agostino & Pearson test and Shapiro–Wilk test. Comparison of groups was subsequently tested with unpaired *t*-tests for normal data sets or Mann–Whitney tests for non-normal data sets.

Tuberous sclerosis lesions were designed and classified considering their histological, immunohistochemical, cytoarchitectural features and their spatial position in brain parenchyma, i.e., ventricular zone (VZ), deep white matter, superficial white matter (SWM) and cortical plate.

## Results

### Clinical Features of TSC Fetus

The present study investigated the brains of 14 fetal cases covering the gestation period 19–39 weeks (GW), one term newborn deceased the 3th postnatal day and one infant case deceased the 8th postnatal month (Table 1). In 25–39GW cases TSC was strongly suspected prenatally after routine echography revealing the presence of presumptive cardiac rhabdomyoma. Subsequent MRI analysis revealed hypersignal in fetal brain, gyration anomalies and periventricular tubers in 34–39GW cases. The 19GW fetus had a familial history of polycystic kidney disease. The 21GW fetus presented an intracerebral mass interpreted as intracerebroventricular hemorrhage. Medical abortion was performed in all fetal cases and anatomopathological analysis of brain samples were performed by AG confirming the diagnosis of TSC. The two postnatal cases deceased from cardiac failure and status epilepticus respectively. Genetic analysis was performed in 8 cases (Table 1) and revealed mutations in 7 cases (case 11 did not display mutations in either TSC1 or TSC2).

### Tuberous Sclerosis Cell Types in Fetal Brains

The histological and cellular features of TSC lesions have been well described in adults and children post-surgery (reviewed in Aronica and Crino, 2014). In the present study we observed quite similar features in 36–39GW TSC brains (see Supplementary Figure 1). However, in earlier gestational ages, the configuration and cellular composition of brain lesions differ according to a developmental evolution that will be described below, notably, the visualization of clear cortical tubers was observed only at 36GW and onward. Interestingly, all the cases displayed subependymal nodules located at the vicinity of the caudate nucleus.

We identified 4 types of cells composing fetal TSC brain lesions, designed as Giant cells, dysmorphic neurons, dysmorphic astrocytes and Small Fusiform (SF) cells.

#### Giant Cells

**FIGURE 1:**
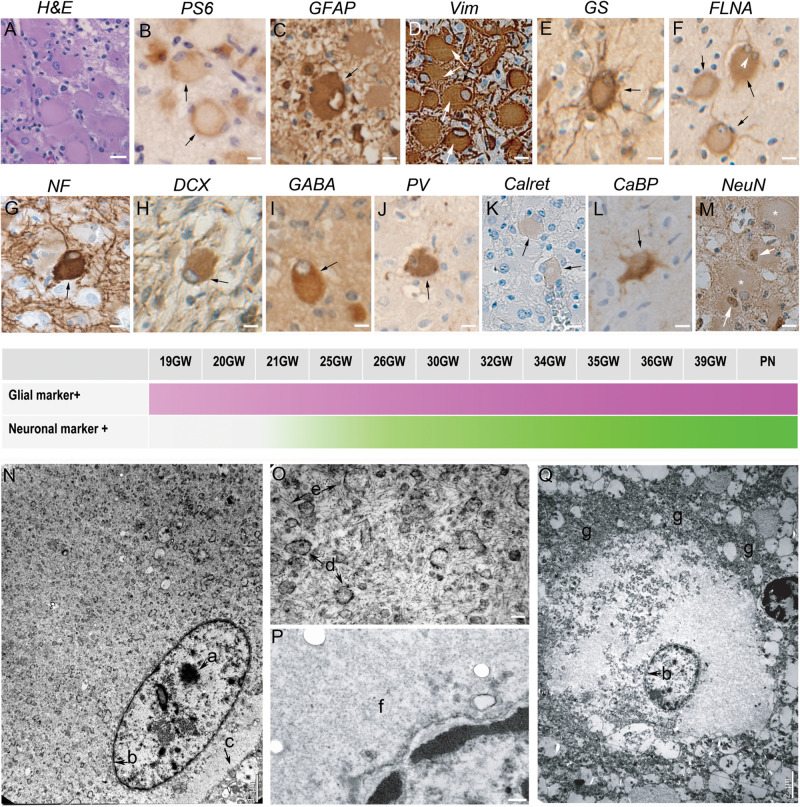
Characterization of Giant cells. **(A)** Hematein-eosin staining of Giant cells displaying eosinophilic cytosols and flattened and decentered nuclei. **(B)** Immunostaining with antibodies to phosphorylated form or ribosomal PS6 (RPS6 R232H) of a couple of Giant cells. **(C)** Glial Fibrillary Acidic Protein immunopositive Giant cell; note the presence of GFAP negative cytomegalic neighboring cells. **(D)** Vimentin immunopositive Giant cell. some thin extensions were also immunopositive. **(E)** Glutamine synthetase antibodies stain the cytosol and the thin extensions of Giant cells. **(F)** Filamin A positive Giant cells, including a bi-nucleated one (arrow head). **(G)** Neurofilament 200 immunopositive balloon-like cell surrounded by immunonegative cytomegalic cells. **(H–M)** Giant cells immunopositive for different neuronal markers: DCX (Doublecortin), GABA (Gamma Amino Butyric Acid), PV (parvalbumin), Calret (Calretinin; only very slightly immunopositive as compared with neighbor cytomegalic cells), CaBP (Calbindin), NeuN (that stains mainly the nuclei). **(N–Q)** Electron microscopy pictures illustrating ultrastructure details of Giant cells displaying either neuronal (**N**, detail of the cytosol enlarged in **O**) or glial (**Q**, detail of the cytosol enlarged in **P**) features. a, nucleolus; b, nuclear membrane; c, cytoplasmic membrane; d, endoplasmic reticulum; e, filaments; f, smooth cytosol; g, accumulation of organelles at the periphery of the cytosol. Arrows in **(A–M)** point to immunopositive Giant cells; * in M indicate NeuN negative Giant cells. Figures were taken from 25GW **(A,D,H,J,K,L)**, 26GW **(B)**, 30GW **(N–Q)**, 34GW **(G,I,M)** and 36GW **(C,E,F)**; from cortex **(E,G,I,N–Q)**, white matter nodule **(A,C,D,J–K,M)** and subependymal nodule **(H)**. Scale bars: 10 μm **(A–M)**, 1 μm **(N)**, 100 nm **(O,P),** and 2 μm **(Q)**. The frieze in the middle schematically represents the intensity of immunostaining of tuberous lesions throughout development. Arrows point to immunopositive Giant cells **(B–M)**.

Figure 1 displayed peculiar and highly recognizable features after hematein-eosin staining. They were cytomegalic round cells with a typical eosinophil smooth cytosol and a small, flattened and decentered nucleus (Figure 1A). Giant cells were immunopositive for PS6 antibodies (Figure 1B), particularly dense at the periphery of the cell, indicating the hyperactivation of mTORC1 pathway. Immunohistochemical analysis indicated the presence of two different subtypes displaying glial or neuronal features. Thus, Giant cells were immunopositive either for glial cell markers: GFAP (Figure 1C), vimentin (Figure 1D) or GS (Figure 1E) or, although less numerous, immunopositive for neuronal markers like NF200 (Figure 1G), DCX (Figure 1H) and even NeuN (Figure 1M), a nuclear antigen expressed normally by mature neurons. More surprisingly, some Giant cells were immunopositive for interneuron markers like GABA (Figure 1I), parvalbumin (Figure 1J), calbindin (Figure 1L) and more scarcely to Calretinin (Figure 1K).

In the cases analyzed here Giant cells were displaying a rather “glial” phenotype and displayed a multipolar aspect with a rounded soma and multiple thin stellar processes; they were also FLNA positive (Figure 1F). Giant cells expressing neuronal markers were observed only after 25GWs in nodules and after 30GWs in cortex (shown schematically in the time graph in Figure 1). They were paucipolar, with rounded soma and sparse neurites. Their nucleus was in some cases rather big with a prominent nucleolus.

Electron microscopy analysis confirmed the presence of Giant cells displaying either neuronal or glial phenotypes accordingly to the nuclear shape (Figures 1N,Q). Neuronal like Giant cells presented a rather large and disorganized cytosol devoid of Golgi apparatus, organized reticulum and microtubules. Bunches of intermediate filaments were abundant around the nucleus (Figure 1O). Glial-like Giant cells displayed a large smooth cytosol devoid of organelles around the nucleus (Figures 1P,Q) and an accumulation of not well-defined organelle pressed at the periphery of the cell, forming a kind of ring of electrodense material (Figure 1Q).

Giant cells were constitutive elements of the TSC lesions associated with the other cell types, or were present outside the TSC lesions, in the cortex or WM. They were frequently grouped into clusters or radial columns (not shown).

#### Dysmorphic Neurons

**FIGURE 2:**
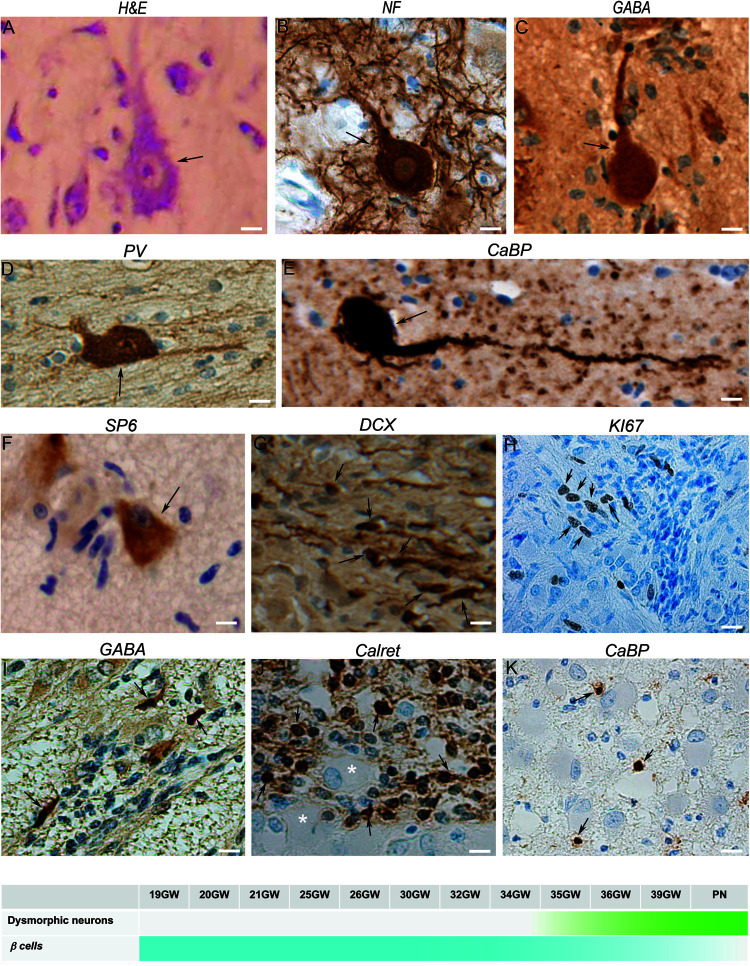
Characterization of neuronal components of TSC lesions. **(A)** Hematein-eosin staining of a typical cytomegalic neuron in a tuber; a few small *SF* cells are aligned at his left. **(B)** Neurofilament 100 immunopositive cytomegalic neuron surrounded by numerous immunopositive extensions in the deep part of a cortical tuber. **(C)** GABA immunopositive cytomegalic neuron surrounded by numerous immunonegative *SF* cells in a white matter nodule. **(D)** Illustrates a parvalbumin positive dysmorphic neuron in a tuber. **(E)** Calbindin + gigantic dysmorphic neuron in a white matter nodule. **(F)** Immunostaining with RPS6 R232H antibodies of a pyramidal like neuron in the cortical subplate. Note that surrounding *SF* cells are immunonegative. **(G)** Doublecortin staining of *SF* cells in a white matter nodule; note the small cell body and the thin tangentially oriented extensions reminiscent of migrating neurons. **(H)** Staining of some *SF* cells in a subependymal nodule with Ki67 antibodies, indicating their proliferative status. **(I)** GABA immunostaining of a few *SF* cells in a subependymal nodule surrounding immunonegative Giant cells (*). **(J)** Calretinin immunostaining of *SF* cells in a deep white matter nodule. **(K)** Staining of some *SF* cells with calbindin antibodies in the core of a white matter nodule. Figures were taken from 21GW **(H)**, 25GW **(I,J)**, 34GW **(G,K)** and 36GW **(A–F)**; from cortex **(A–D,F)**, white matter nodule **(G–K)** and subependymal nodule **(E)**. Arrows point to cell bodies of some immunopositive cells. Scale bars: 10 μm. The frieze in the bottom schematically represents the intensity of immunostaining of tuberous lesions throughout development.

Figure 2 were cytomegalic as previously described (Aronica and Crino, 2014). In hematein-eosin staining they displayed a triangular or oval soma containing Nissl bodies, a big nucleus, sometimes decentered, and a well discernable nucleolus (Figure 2A). They were immunoreactive to PS6 (Figure 2F) and were positive for neuronal markers like NF200 (Figure 2B), DCX and NeuN (not shown). In fetal cortical and subcortical tubers, we observed some dysmorphic neurons immunopositive for interneuron markers, mostly GABA (Figure 2C), parvalbumin (Figure 2D) and calbindin (Figure 2E) and occasionally to Calretinin (not shown).

Dysmorphic neurons were first detected at 34GW in subcortical nodules and at 36GW in cortical tubers (timeline graph in Figure 2). They were also observed in a dispersed form in the cortex outside TSC lesions.

#### Dysmorphic Astrocytes

**FIGURE 3:**
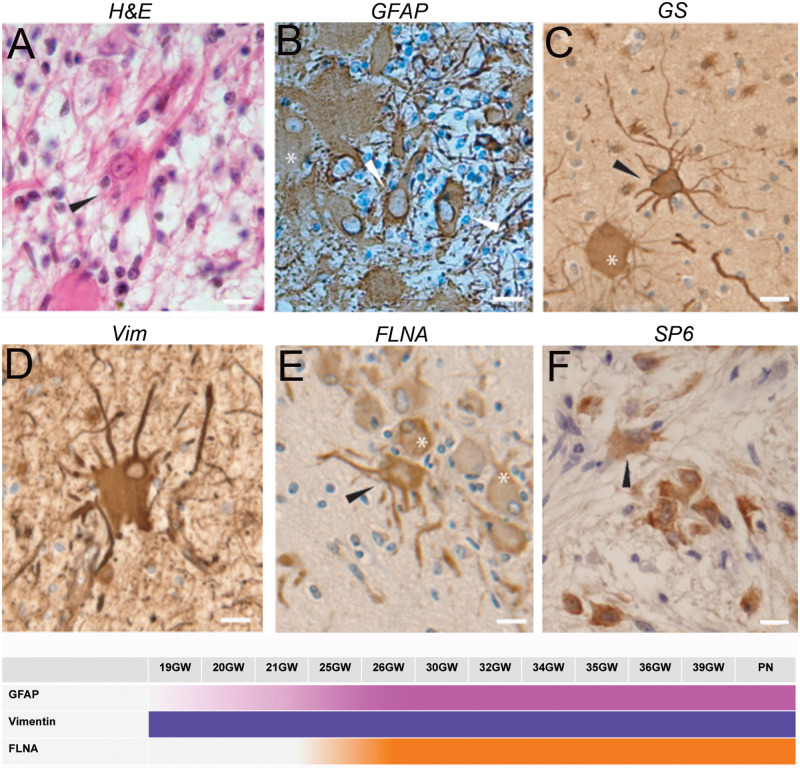
Characterization of dysmorphic astrocytes. **(A)** Hematein-eosin staining of a cytomegalic astrocyte (arrowhead) in a white matter nodule displaying a long thick extension. Also note the presence of numerous *SF* cells. **(B)** GFAP immunostaining of astrocytes (arrowheads) and Giant cell (^∗^). **(C)** Glutamine synthetase staining of a Giant cell (^∗^) and a dysplastic astrocyte (arrowhead) in a tuber. Note that astrocytic extensions were thicker and longer than that of Giant cells. **(D)** Vimentin immunostaining of a dysmorphic cytomegalic astrocyte in a white matter nodule. **(E)** Filamin A immunostaining of an astrocyte (arrow head) and some Giant cells (^∗^) in the superficial white matter. **(F)** Immunostaining with RPS6 R232H of glial cells in a subependymal nodule. Arrowhead points to a dysmorphic astrocyte. Pictures were taken from 25GW **(B)**, 30 **(F)**, 34GW **(A)** and 36GW **(C–E)**; from white matter nodules **(C–E)** and subependymal nodules **(A,B,F)**. Scale bars: 10 μm. The frieze schematically represents the intensity of immunostaining of tuberous lesions throughout development.

Figure 3 as stained by hematein-eosin presented a large eosinophilic cell body with not well-delimited borders and a tortuous shape. Numerous extensions arose from their somas. In most of the cases they also displayed a rather long and thick-branched extension (Figure 3A). Dysmorphic astrocytes were immunopositive for the same markers as Giant cells: GFAP (Figure 3B), GS (Figure 3C), vimentin (Figure 3D), filamin (Figure 3E) and commonly PS6 (Figure 3F) but differed from them by the shape of their body and nucleus, generally centered; they differed from typical reactive astrocytes by their larger soma and the presence of thick and tortuous extensions. In subependymal nodules astrocytes acquired “gemistocytic” features: large cytoplasmic mass, long branching processes, and increased cytoplasmic filaments.

Dysmorphic astrocytes were observed in every fetal case and all TSC lesions. They were particularly abundant in subependymal nodules. They were also immunopositive for FLNA (Figure 3E) in 25–39GW cases but not before (see the time graph in Figure 3).

#### Small Fusiform (SF) Cells

Figure 2 were small fusiform basophilic cells, always present in TSC lesions. They did not present dysmorphic features, furthermore, with few exceptions, they were PS6 immunonegative (Figure 2F). These cells would be germinal matrix cells (see Prabowo et al., 2013), though less than 5% of them were Ki67 immunopositive (a cellular marker for proliferation Figure 2H), and post-mitotic migrating neurons as most of them were immuno-positive for DCX antibodies and displayed typical features of migrating neurons (Figure 2G). Interestingly, many *SF cells* were immunopositive for GABA, calretinin and calbindin (Figures 2I–K), occasionally for parvalbumin, suggesting that a large proportion of them are migrating interneurons. Although *SF* cells are normal cells they were quite abundant in white matter and subependymal nodules, where they formed fusiform flows of cells. In cortical plate lesions, they form sparse rounded clusters (not shown).

#### SF Cells

SF cells were observed for the first time at 19GW in deep WM and subependymal nodules, at 25GW in the superficial WM. Calretinin + *SF cells* were observed at 25GW and calbindin + at 34GW.

### TSC Developmental Changes

#### Development of Subependymal Nodules

**FIGURE 4:**
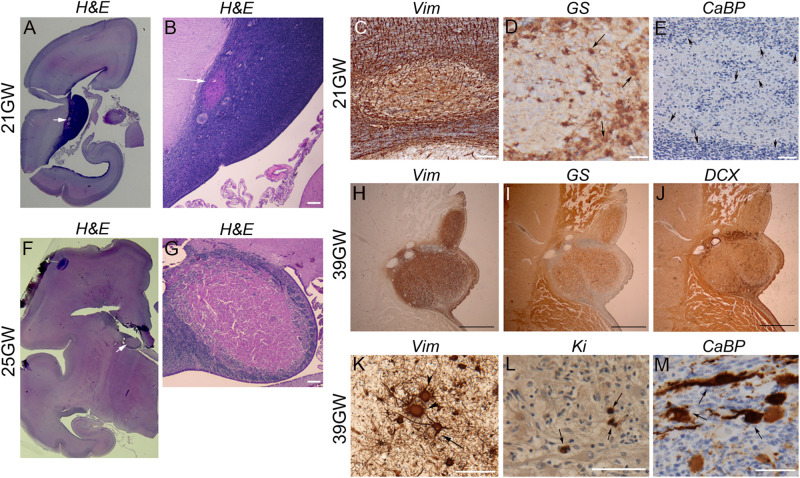
Development of Subependymal Nodules. **(A–E)** Subependymal nodules were detected at 21GW within the ventricular subventricular zone. As seen in H&E staining **(A,B)** small nodule (arrows) was disrupting the germ cell layer, disorganizing the radial arrangement of radial glial cells as seen with vimentin antibodies **(C)**. Some GS + Giant cells (arrows) and dysmorphic astrocytes were already populating the tumor though they accumulate more at the periphery **(D)**. CaBP antibodies **(E)** did not stain subependymal nodule cells; rather note the presence of small fusiform cells (arrows depict some of them) stained with Nissl at the periphery and at the heart of the nodule. **(F,G)** Images of a larger nodule taken from 25GW case. It makes clear protrusion within the ventricle (arrow in **F**) acquiring a rosacea configuration, with a large core of eosinophilic cells (mainly gemistocytic astrocytes) and an external ring of *SF* cells **(G)**. **(H–M)** Subependymal nodule from 39GW case. Astrocytes (arrow in K) and Giant cells (arrow heads in **K**) in Subependymal nodules were immunopositive for vimentin **(H,K)** and glutamine synthetase + **(I)**. *Small fusiform* cells can be displaced progenitors (some are indeed Ki67 immunopositive as seen (arrows) in **L**) but the majority were DCX + **(J)** suggesting that they are migrating neurons. Some dysmorphic cytomegalic (arrows) calbindin + cells can be observed on the external ring of the subependymal nodule **(M)**. Figures were taken from 21GW **(A–E)**, 25GW **(F,G),** and 39GW **(H–M)**. Scale bars: 1 mm **(B,G)**, 100 μm **(C–E)**, 500 μm **(H–J),** and 100 μm **(K–M)**.

They were observed in the brain sections from 19 to 21GW when they were characterized by small disruptions of the germinal layer (Figures 4A,B) with interruption of the typical vimentin positive radial glial extensions (Figure 4C). The nodules at these stages were primarily composed of dysmorphic astrocytes, although at 21GW some Giant cells might be present at the periphery of the nodule (Figure 4D). *Small fusiform* cells form small clusters within the core of the nodule (Figure 4E). Immunostaining with neuronal markers (e.g., calbindin in Figure 4E) did not reveal the presence of positive cells in these nodules at this stage. Bigger nodules making protrusion into the ventricle were observed at 24GW and onward (Figures 4F,G); they were characterized by the increased number of dysmorphic astrocytes and Giant cells of glial type (Figures 4G–K). Only a few cells in subependymal nodules were immunopositive for Ki67 antibodies (Figure 4L). By 25GW, a ring of DCX + *SF* cells (Figure 4J), some of them calbindin + and calretinin + were observed in these nodules in all the cases analyzed. In samples 25GW and onward, dysmorphic, cytomegalic neurons calbindin + (Figure 4M) were also observed at this external ring in every case.

#### Development of White Matter Nodules

**FIGURE 5:**
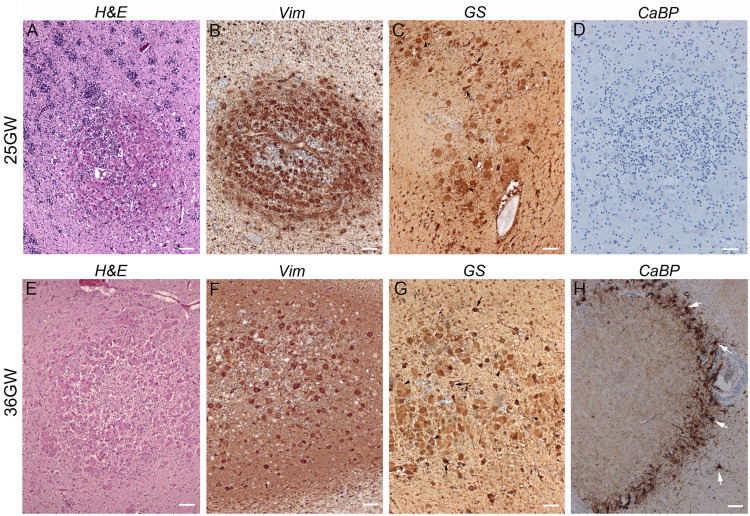
Development of white matter nodules. **(A–D)** At 25GW hematein-eosin **(A)**, vimentin **(B)**, glutamine synthetase **(C),** and Calbindin **(D)** staining revealed the presence of Giant cells and dysmorphic astrocytes in white matter nodules. At this stage numerous *SF* cells distribute within the core of the nodule and at the periphery (as seen thanks to blue Nissl counterstaining), intermingled with cytomegalic cells. None cells were immunopositive for Calbindin **(D)**. **(E–H)** At 36GW the nodule was less enriched in vimentin + **(F)** and GS + Giant cells **(G)**, tend to be organized around a core of *SF* cells and displayed a sharp external “ring” of *SF* cells and dysmorphic neurons (white arrows) both calbindin + **(H)**. Arrowheads and arrows in **(C,G)** point to some Giant cells and dysmorphic astrocytes respectively. Scale bars: 100 μm.

Present in the deep part of the WM, corresponding to the intermediate zone and the SVZ, from 19GW onward and localized also in the superficial WM (subplate) from 25GW. These nodules increased in size during development likely due to an increase in the number of Giant cells. At 19–21GW (Figures 5A–D) WM nodules were composed by DCX (+) SF cells particularly enriched in the core of the nodule, and vimentin + (Figure 5B) or GS + (Figure 5C) Giant cells and dysmorphic astrocytes. The density of vimentin positive cells was particularly striking at these stages. From 30 to 39 GW (Figures 5E–H) WM nodules were also enriched in Giant cells and dysmorphic astrocytes (Figure 5E) immunopositive for vimentin (Figure 5F) and GS (Figure 5G) and included also, from 26 to 34GW, DCX + Giant cells. In addition, from 34GW, dysmorphic neurons some of them calbindin + (Figure 5H) and occasionally parvalbumin + (not shown) were observed. Notably, nodules localized in deep white matter were surrounded by a ring of calbindin + cells, some of them dysmorphic at 34GW and onward (Figure 5H).

#### Development of Cortical Tubers

**FIGURE 6:**
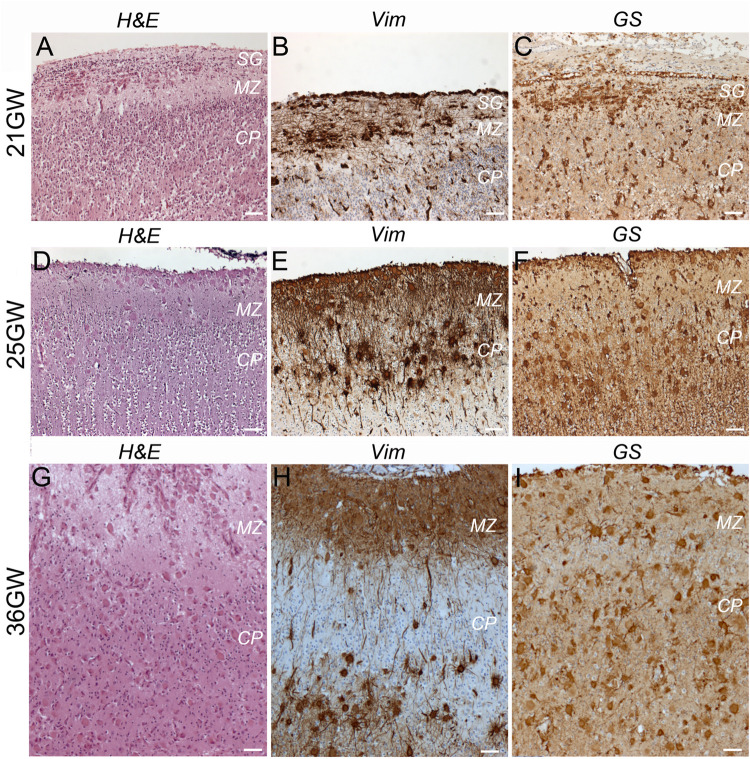
Development of cortical TSC lesions: dysmorphic astrocytes and Giant cells. **(A–C)** At 21GW Hematein-eosin (H&E) staining and immunolabelings with vimentin (Vim) and Glutamine synthetase (GS) antibodies revealed the presence of dysmorphic astrocytes in the subpial granular layer (*SG*). **(D–F)** At 25GW numerous Giant cells can be visualized in the subpial granule cell layer, marginal zone and cortical plate, immunopositive for Vim and GS. **(G–I)** at 36GW cortical lesions evolve with Giant cells (Vim + and GS + −) being also present in the subplate. SG, subpial granular layer, MZ, marginal zone, CP, cortical plate. Scale bars: 100 μm.

**FIGURE 7:**
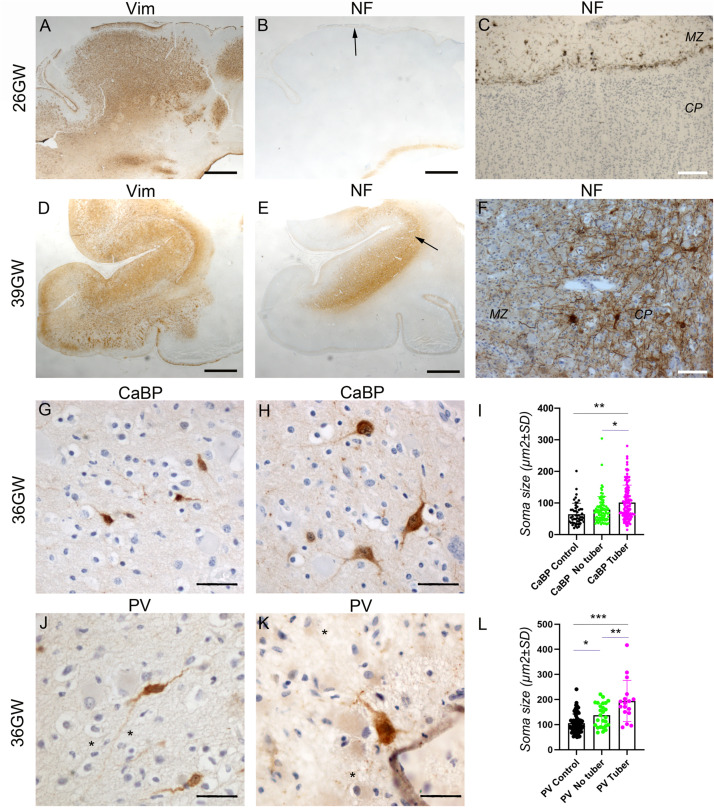
Development of cortical TSC lesions: Neuronal features. **(A–C)** Pre-tuberal lesion on the cortex of a 26GW case, involving the presence of multiple vimentin positive Giant cells and dysmorphic astrocytes. However, NF200 immunopositive dysmorphic neurons are absent **(B,C)**. Only the normal Brun layer of tangential fibers appears immunopositive in the marginal zone **(C)**. **(A,B)** are close sections from the same cortex. The area depicted by arrow in **(B)** is enlarged in **(C)**. **(D–F)** A typical tuber from a 39 GW case displaying an intense vimentin staining of Giant cells and dysmorphic astrocytes **(D)** and prominent NF200 immunostaining **(E,F)** of dysmorphic neurons and bundles of neurites, which accumulate mainly in the deep part of the tuber. **(D,E)** are close sections from the same cortex. The area depicted by arrow in **(E)** is enlarged in **(F)**. **(G–I)** Calbindin immunostaining of cortex from a 36GW case. In non-tuberal cortex **(G)** the majority of immunopositive cells are of small size and display immature features. In tubers **(H)** frequent cytomegalic or dysmorphic calbindin positive cells were observed. **(I)** Quantification of cell soma size of stained neurons from 36 to 37GW control and TSC cases. Note that mean soma size is increased in tubers, with numerous cells displaying rater big soma size (*n* = 51 (control), 87 (no tuber) and 130 (tuber) cells). * and **p = 0.012 and 0.0004, respectively (Mann–Whitney test). **(J–L)** Parvalbumin immunostaining of cortex from a 36GW case. In non-tuberal cortex **(J)** the majority of immunopositive cells are of small size and display immature features. In tubers **(K)** a few cytomegalic or dysmorphic PV positive cells were observed (* tags Giant cells immunonegative). **(L)** Quantification of cell soma size of stained neurons from 36 to 37GW control and TSC cases. Note that mean size is increased in tubers, with some cells displaying rather big soma size (*n* = 63 (control), 26 (no tuber) and 17 (tuber) cells). *, **, and *** *p* = 0.036, 0.0013, and 0.0001 respectively (*t*-test). SG, subpial granular layer, MZ, marginal zone, CP, cortical plate. Scale bars: 2 mm **(A,B,D,E)**, 100 μm **(C,F)**, and 50 μm **(G,H,J,K)**.

TSC alterations at the cortical level are early events already present at 21GW (Figure 6). However, the typical cortical tuber features, as those described in children and adult patients (i.e., malformations of the cerebral cortex presenting as a focal thickening of the cortex, involving the presence of densely packed dysmorphic neurons and Giant cells and the loss of the normal cortical laminar organization), were only observed at 36–39GW (Figures 6, 7 and Supplementary Figure S1).

At 21GW we observed the presence of dysmorphic astrocytes (Figures 6A–C) Vim+, GS+, and GFAP+; they were restricted to the marginal zone and subpial granule cell layer where they adopt a tangential orientation. In this layer hematein-eosin staining revealed the presence of some *SF cells*, but they were immunonegative to all markers tested. Giant cells were not yet observed either in the marginal zone or in the cortical plate. At this stage no sign of dysplasia could be observed at the cortical plate whose lamination appeared unaltered.

At 25–34GW (Figures 6D–F, 7A) both Giant cells and dysmorphic astrocytes, Vim+, GS+, and GFAP + were observed distributed along the marginal zone and invading also the more superficial part of the cortical plate (presumptive layer L2; Figures 6D–F). They were also observed in the deep part of the cortex (presumptive layers LV-LVI). The presence of dysmorphic cells in the cortical plate appears to disrupt the normal lamination. These developmental changes are compatible with the notion that Giant cells and dysmorphic astrocytes invade the cortical tuber from two different origins, the subpial granule cell layer and the white matter. At these stages dysmorphic neurons were not observed yet and NF200 antibodies decorated only a thin band of tangential fibers in this marginal zone (Figures 7B,C). This NF200 staining was similar to that observed in patients’ areas free of TSC lesions or in age-matched control cortex.

At 36–39 GWs (Figures 6G–I, 7D), Giant cells and dysmorphic astrocytes immunopositive for vimentin, GS and GFAP were present in the cortical plate, accumulating particularly at the superficial and deep cortical layers (Figure 6H). The more significant change observed at 36GWs as compared with earlier stages is the appearance of NF200 + dysmorphic neurons (183–240 cells per cortical tuber section; mean density was 8.65 ± 2.54/mm^2^); they were more enriched in the deeper part of the tuber and created an exuberant band of neurofilament + neurites (Figures 7E,F), while the apparently normal cortex surrounding the tuber was almost devoid of NF staining, with the exception of the staining of tangential fibers in the molecular layer that is a normal feature of human developing cortex (Bystron et al., 2008).

It is interesting to note that dysmorphic neurons immunopositive for interneuron markers were also found in tubers. Though their numbers were relatively modest (6–14 calbindin + and 1–4 parvalbumin + cells per 36GW tuber section) they represented an important proportion of their respective population (20.7% of total calbindin + cells and 23.5% of total parvalbumin + cells in tubers). These cells presented with abnormal nuclei or abnormal soma shape and/or rather big soma size (see Figures 7H,K) as compared with cells in adjacent non-tuberal cortex (Figures 7G,J) or the cortex of a three control 36–37GW fetuses. They also displayed a more developed dendritic compartment. Furthermore, quantitative analysis of all calbindin + or parvalbumin + cells (Figures 7I,L) indicates that mean soma size was higher in the tuber than in non-tuberal cortex from the same fetal cases or in control cortex from unaffected fetuses. It is important to note, dysmorphic interneuron-like cells were observed in non-tuberal areas of affected individuals however that they accounted for only 2.7% of calbindin + and 5.8% of parvalbumin + cells.

## Discussion

**FIGURE 8:**
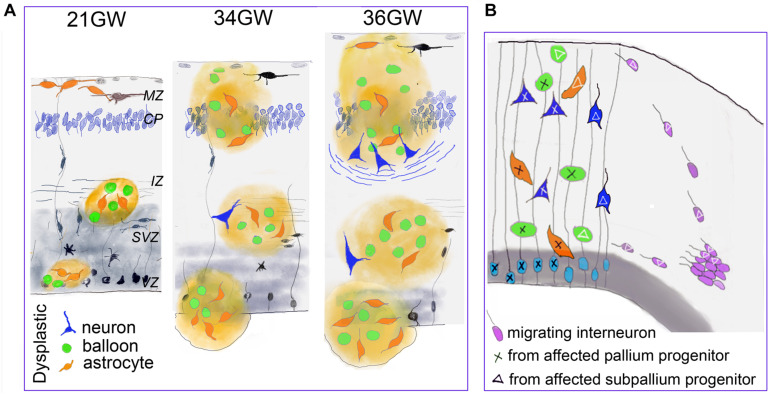
Developmental changes of TSC lesions. **(A)** Schematic representation of the main changes observed in fetal TSC lesions. Dysmorphic cells are represented in green (Giant cells) orange (dysmorphic astrocytes) and blue (dysmorphic neurons). MZ, marginal zone; CP, cortical plate; IZ, intermediate zone; SVZ, subventricular zone; VZ, ventricular zone. **(B)** Schematic representations suggesting that TSC lesions would be mosaic and result for the development of two different neural progenitors: from pallium (light blue) and subpallium (violet). This hypothesis is supported by our data indicating that a subgroup of dysmorphic neurons and Giant cells express markers of GABA interneurons and that part of tuber cells would enter from the marginal zone. As only some cells display dysplastic features, we speculate that TSC mutation impact the developmental program of subset of cell lines (tagged with X or triangle).

The present data indicate that the cellular composition, the cytoarchitectonic organization and the distribution of TSC cells and lesions clearly evolve during fetal development (schematized in Figure 8A) confirming the progressive character of TSC lesions during pregnancy.

In agreement with previous reports (Bordarier et al., 1994; Park et al., 1997; Prabowo et al., 2013) we confirm the early appearance of TSC lesions, characterized in our 19–21GW samples by white matter and subependymal nodules inclosing dysmorphic astrocytes, GS + Giant cells and *SF* cells. At 21GW, the cortical plate contained already some dysmorphic astrocytes in the marginal zone, but they are not organized to form a tuber. Giant cells were not observed in the cortex at this stage and neuronal lamination seems unaffected. Therefore, the genesis of dysmorphic astrocytes and Giant cells and their presence in subcortical zones seem to be the initial cellular events in TSC. Previous studies already identified the presence of both reactive and dysmorphic astrocytes immunopositive for GFAP and vimentin in cortical tuberal lesions (Boer et al., 2008a, b, 2010; Sosunov et al., 2008) and *in vitro* studies on developing human cortical spheroids that mTORC1 hyperactivation results in a greater production of astrocytes at the expense of neurons (Blair et al., 2018).

The presence of dysmorphic neurons is a relatively late event on TSC lesion development. They were first observed by 34GW in subcortical nodules and only at 36–39GW in cortex. Therefore, the typical cortical tubers as commonly defined in the literature (i.e., containing Giant cells and dysmorphic neurons), were only detected from 36GW. Interestingly, in our samples, the apparition of well delineated cortical tubers is coincident with the appearance of dysmorphic neurons and neurofilament positive bundles of fibers that accumulate at the bottom of the tuber and which might help the identification of cortical tubers on medical imaging studies and brain sections examined macroscopically.

Previous immunohistochemical analyses of FCD samples with markers of progenitor types (Lamparello et al., 2007) strongly suggested that balloon cells derived from pallial radial glial cells, as subsets of balloon cells express markers of radial glial progenitors like MASH1 or PAX6. These data are reinforced by our observations that lesions observed in samples from 19 to 21GW localize in cortical germinative zones (VZ and SVZ) where the typical radial arrangement of these progenitors is disrupted. Thus, dysmorphic cells originating from VZ/SVZ would migrate to the intermediate zone/white matter to form TSC lesions first in the intermediate zone, later one in the subplate and finally in the cortical plate. In agreement with this, Mühlebner et al. (2016) found on surgically resected cortical tubers, that many dysmorphic neurons were positive for different cortical layer markers (mainly Cux2, ER81, and RORß), thus resembling cortical projection neurons. However, our data strongly suggest that dysmorphic neurons and Giant cells might have a dual origin, the pallium and the subpallium: (i) Our data suggest that some Giant cells invade the cortical plate (upper cortical layers) migrating (either differentiated or immature) from the subpial granule cell layer and marginal zone by 25GW, while many others enter into the cortical plate migrating apparently from white matter by 26–30GW. It is important to remember that in the developing human brain and non-human primates the marginal zone (future layer 1) and the transient subpial granule cell layer contain cells generated in the cortical hem and subpallium (Cajal Retzius cells and interneurons; Gadisseux et al., 1992; Meyer and Goffinet, 1998; Marin and Rubenstein, 2001; Zecevic and Rakic, 2001; Bielle et al., 2005; García-Moreno et al., 2007; Ceci et al., 2012), and that interneurons migrating in the marginal zone penetrate and colonize the underlying cortical plate. Furthermore, in primates, a protracted genesis of marginal zone/layer 1 GABA cells, until the end of corticogenesis, has been described (Zecevic and Rakic, 2001). (ii) We consistently observed that interneuron markers such as GABA, calbindin and parvalbumin stained subset of dysmorphic neurons and Giant cells, both in cortical and subcortical TSC lesions. This suggests that certain interneuron progenitors are also affected by TSC mutations and contribute to the development of TSC lesions (schematized in Figure 8B). This observation is in contrast with recent data showing in a conditional mouse line, that an abnormal increased activation of the mTOR pathway in interneuron lineage only was not sufficient to generate a cortical dysplasia (D’Gama et al., 2017) and it was then concluded that dysplastic lesions required the involvement of projection neuron progenitors. In agreement with our findings, samples from patients with Focal Cortical Dysplasia, which display many cellular similarities with TSC lesions, contain a small subset of dysmorphic neurons that express VGAT and DLX1 or DLX2 transcription factors expressed by cells derived from the medial ganglionic eminences (Lamparello et al., 2007). These observations with our present data, particularly the high proportion of parvalbumin and calbindin positive cells that were dysmorphic and cytomegalic in cortical tubers, tend to suggest that TSC lesions are mosaics lesions generated from different classes of progenitors, from pallium and subpallium, a feature that should not be surprising for a multisystemic pathology like TSC originated in most of the reported cases by germ-line TSC2 or TSC1 mutations.

One intriguing component of TSC lesions is the group o*f SF cells*, particularly abundant in subependymal and white matter nodules. These cells are more likely to be post-mitotic/migrating neurons, which are abundant on the areas where these lesions develop. Indeed, a large majority of *SF cells* are DCX positive, displaying typical morphological features of migrating neurons. In our samples *SF cells*, with few exceptions, were pS6 negative, suggesting that in these cells or during their immature developmental status, the mutation of TSC genes have no impact on their biology. However, it cannot be excluded that changes in the phosphorylation of this protein remain undetectable because of the narrowness of their cytosolic compartment. It is important to highlight that many of these “immature” cells fails to migrate and persists ectopically in subcortical areas, even in postnatal patients. This alteration depends either on cellular autonomous mechanisms or is the consequence of the presence of TSC subcortical nodules, which would impact their migratory behavior by a non-cellular autonomous mechanism. In SEN, the emergence of Giant cells and gemistocytic astrocytes seems to push *SF cells* to the periphery, where they will delineate an external ring giving the nodules the appearance of a rosacea. In WMNs *SF* cells were in contrast more enriched at the core of the lesion mixing with Giant cells and dysmorphic astrocytes.

Subsets of *SF cells* are by 25GW immunopositive for GABA, calretinin and calbindin. Two main hypothesis can explain the presence of these putative migrating interneurons in the nodules: first, in our samples subependymal nodules were localized at the caudothalamic groove, that can be considered as a corridor for migrating interneurons generated in the ganglionic eminences; these migrating interneurons would penetrate or circumvent the nodule and enwrap its central core of Giant cells and dysmorphic astrocytes. Second, at midterm (by around 20GW) a subset of cortical calretinin + cells proliferate in the cortical subventricular zone (SVZ) (Zecevic et al., 2011), where subependymal nodules initiate their formation. It is interesting to note that dysmorphic neurons immunopositive for calbindin or parvalbumin were frequently mixed with *SF cells* contributing to the formation of an external ring of calbindin or parvalbumin positive cells, in both SEN and WMN. It is possible that these dysmorphic interneuron-like cells derive from *SF cells.*

The first detected alteration in TSC fetus is cardiac rhabdomyoma, a benign fetal cardiac tumor commonly associated with TSC (Bader et al., 2003; Saada et al., 2009), and this was the case for the majority of the cases investigated here. With the development of high-resolution ultrasound echocardiography and magnetic resonance, the prenatal diagnosis in the second and third trimesters of pregnancy increased considerably (Bejiqi et al., 2017; Mariscal-Mendizábal et al., 2019). Prenatal diagnosis has gained interest with recent data suggesting that maternal treatment with inhibitors of the mTOR pathway (everolimus and sirolimus) may be beneficial for rhabdomyomas without any serious adverse events reported (Barnes et al., 2018). It is likely that early treatment during pregnancy could also prevent the development of brain TSC lesions described here, precluding the development of clinical manifestations associated with them like epilepsy and cognitive alterations. Indeed, several studies have shown encouraging results in children with TSC treated with these compounds (Bevacqua et al., 2019; Saffari et al., 2019), in particular a reduction in the size of lesions and the frequency of seizures. Our data demonstrating progression of TSC lesions during fetal life and support the importance of earlier diagnosis and treatment with mTOR inhibitors for the prevention of brain damage and associated clinical manifestations.

## Data Availability Statement

All datasets presented in this study are included in the article/Supplementary Material.

## Ethics Statement

Ethical review and approval was not required for the study on human participants in accordance with the local legislation and institutional requirements. Written informed consent to participate in this study was provided by the participants’ legal guardian/next of kin.

## Author Contributions

AG and AR performed the analysis, acquired pictures, interpreted the observations and data, and wrote the manuscript. Both authors contributed to the article and approved the submitted version.

## Conflict of Interest

The authors declare that the research was conducted in the absence of any commercial or financial relationships that could be construed as a potential conflict of interest.

## Supplementary Material

FIGURE S1 Characterization of TSC lesions in 36–39 GW cases. **(A)** Nissl staining of a coronal section from 36GW illustrating the localization of three types of lesions: Tuber (T), white matter nodule (WmN) and Subependymal nodule (SE). **(B–D)** Cortical Tuber from 39GW case (also illustrated in Figures 7D,E) stained with hematein-eosin (H&E), Glutamine synthetase (GS) and neurofilament 200 NF. *Cortical tubers* were defined by a focal thickness of the cortical ribbon with a clear-cut delimitation from surrounding cortex and WM. Unlike the adjacent cortex, the tuber was devoid of radial or layered organization of cells **(B)**. The tuber contained densely packed and intermingled normal neurons, Giant cells mainly GS + **(C)**, dysmorphic neurons mainly NF + **(D)**, which were mainly concentrated in the deep part of the tuber, dysmorphic astrocytes and *SF* cells that were more disseminated. **(E–H)** White matter nodule from 36GW case stained with H&E, vimentin (Vim), GS and calretinin. *White matter nodules* were rounded or elliptic formations containing cells organized at least into 2 concentric layers. They displayed a core of *SF* cells, Giant cells and dysmorphic astrocytes positive for vimentin, GS, GFAP and an external ring of *SF* cells positive for calretinin (FH) or calbindin antibodies that were intermingled with Giant cells. Also, depending of term, dysmorphic neurons were present in the nodule. Nodules localized either in the deep part of the cortical mantle (corresponding to the subventricular zone) and the intermediate zone (the deep white matter) or were more superficial and localized in the subplate (superficial with matter). **(I–L)** Subependymal nodule from 36GW case stained with H&E, Vim, GS and calbindin (CaBP). *Subependymal nodules* were observed in all cases evaluated in this study present at the caudothalamic groove, an important landmark when performing neonatal cranial ultrasound. Like SEGA, these nodules were composed mainly by “gemistocytic” astrocytic cells displaying large cytoplasmic mass, long, branching processes, and increased cytoplasmic filaments that were immunopositive to GFAP and vimentin **(J)**. GS + Giant cells were also present in these lesions **(K)**. Only a few numbers of proliferating cells were observed within the nodules (less than 3% of KI67 immunopositive cells). The external part of subependymal nodules contained important numbers of *SF* cells, some of them DCX, calbindin **(L)** or calretinin positive. These nodules localized in the germinative zone and made protrusion into the ventricle. Scale bars: 100 μm **(B,E–L)** and 10 mm **(C,D)**.

## Abbreviations

GW: gestation week
MRI: magnetic resonance imaging
ND: not determined
PD: postnatal day
PM: postnatal month
SEN: subependymal nodule
US: diagnostic ultrasound
WMN: white matter nodule.
