# Supplementary figures and images for: Progression of Fetal Brain Lesions in Tuberous Sclerosis Complex
Source: Front Neurosci. 2020 Aug 21;14:899. doi: 10.3389/fnins.2020.00899 (PMC7472962; doi:10.3389/fnins.2020.00899)

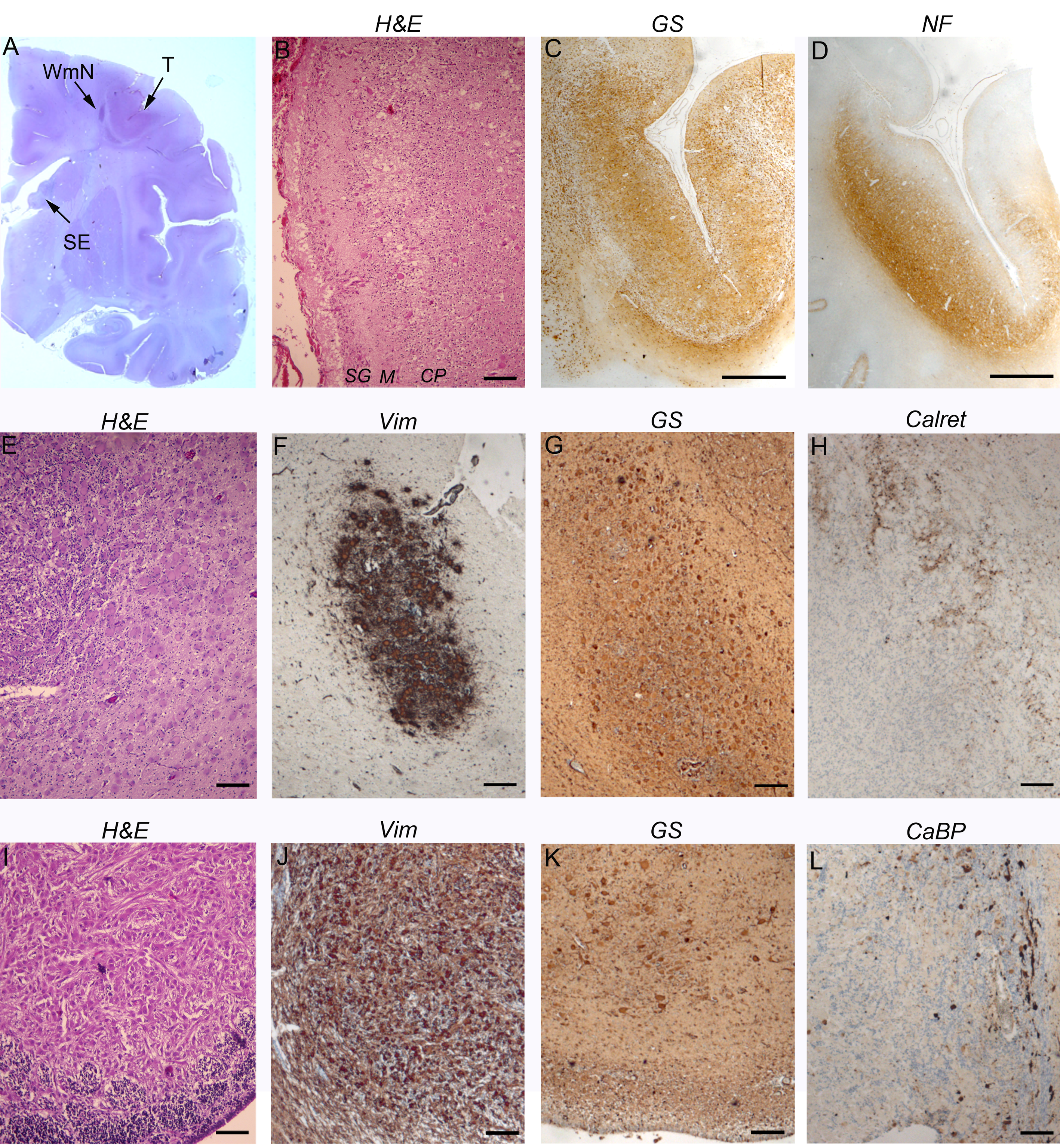

Supplement: FIGURE S1 — Characterization of TSC lesions in 36–39 GW cases. (A) Nissl staining of a coronal section from 36GW illustrating the localization of three types of lesions: Tuber (T), white matter nodule (WmN) and Subependymal nodule (SE). (B–D) Cortical Tuber from 39GW case (also illustrated in Figures 7D,E) stained with hematein-eosin (H&E), Glutamine synthetase (GS) and neurofilament 200 NF. Cortical tubers were defined by a focal thickness of the cortical ribbon with a clear-cut delimitation from surrounding cortex and WM. Unlike the adjacent cortex, the tuber was devoid of radial or layered organization of cells (B). The tuber contained densely packed and intermingled normal neurons, Giant cells mainly GS + (C), dysmorphic neurons mainly NF + (D), which were mainly concentrated in the deep part of the tuber, dysmorphic astrocytes and SF cells that were more disseminated. (E–H) White matter nodule from 36GW case stained with H&E, vimentin (Vim), GS and calretinin. White matter nodules were rounded or elliptic formations containing cells organized at least into 2 concentric layers. They displayed a core of SF cells, Giant cells and dysmorphic astrocytes positive for vimentin, GS, GFAP and an external ring of SF cells positive for calretinin (FH) or calbindin antibodies that were intermingled with Giant cells. Also, depending of term, dysmorphic neurons were present in the nodule. Nodules localized either in the deep part of the cortical mantle (corresponding to the subventricular zone) and the intermediate zone (the deep white matter) or were more superficial and localized in the subplate (superficial with matter). (I–L) Subependymal nodule from 36GW case stained with H&E, Vim, GS and calbindin (CaBP). Subependymal nodules were observed in all cases evaluated in this study present at the caudothalamic groove, an important landmark when performing neonatal cranial ultrasound. Like SEGA, these nodules were composed mainly by “gemistocytic” astrocytic cells displaying large cytopl [file Image_1.TIF]
