# Supplementary material for: Progression of Fetal Brain Lesions in Tuberous Sclerosis Complex
Source: Front Neurosci. 2020 Aug 21;14:899. doi: 10.3389/fnins.2020.00899 (PMC7472962; doi:10.3389/fnins.2020.00899)
Supplement: Supplementary file 2 [file Table_1.doc]

**Supplemental Table 1:** list of antibodies used in this study

| **Antibody** | **Reference** | **Concentration** | **Patients** |
| --- | --- | --- | --- |
| *Mouse anti*-**Calbindin** | Leica Microsystem, clone KR6 | 1:100 | 2,3,5,6,9,10,11,12,13,14 |
| *Mouse anti-***Calretinin** | RTU Leica Microsystem, clone CAL6 | RTU Leica Microsystem | 2,3,5,6,9,10,11,12,13,14 |
| *Goat polyclonal anti-***Doublecortin** (DCX) | Abcam, ab113435 | 1:600 | 2,3,5,6,9,10,11,12,13,14 |
| *Rabbit anti*-**FLNA** | Abcam, clone EP2405Y, ab76289 | 1:250 | 2,3,5,6,9,10,11,12,13,14 |
| *Rabbit  anti-***GABA** | Sigma, A2052 | 1:500 | 2,3,6,10,14 |
| *Mouse anti-***GFAP** | RTU Leica Microsystem, clone GA5 | RTU Leica Microsystem | 2,3,5,6,9,10,11,12,13,14 |
| *Rabbit anti-***Glutamine Synthetase** (GS) | Abcam, ab49873 | 1:5000 | 2,3,5,9,10,11,12,13,14 |
| Mouse anti-**Ki67** | RTU Leica Microsystem, clone MM1 | RTU Leica Microsystem | 2,3,6,10,14 |
| *Mouse* anti-**NeuN** | Millipore,  clone A60, MAB377 | 1:50 | 2,3,6,10,14 |
| *Mouse anti*-**NF200** | Leica Biosystem, clone N52.1.7, PA0371 | RTU Leica Microsystem | 2,3,5,6,9,10,11,12,13,14 |
| *Sheep anti*-**Parvalbumin**. | R&D systems,  AF5058 | 1:320 | 2,3,5,6,9,10,11,12,13,14 |
| *Rabbit anti*-**PS6** | Cell Signaling Technology, clone 4857 | 1:100 | 2,3,5,6,13,14 |
| *Mouse anti-***Reelin** | Chemicon, clone G10, MAB5364 | 1:400 | 2,3,5,6,9,10,11,12,13,14 |
| *Mouse anti-***Vimentin** | RTU Leica Microsystem, clone V9 | RTU Leica Microsystem | 2,3,5,6,9,10,11,12,13,14 |
